# The Influence of DNA Extraction Procedure and Primer Set on the Bacterial Community Analysis by Pyrosequencing of Barcoded 16S rRNA Gene Amplicons

**DOI:** 10.1155/2014/548683

**Published:** 2014-07-10

**Authors:** Ingo C. Starke, Wilfried Vahjen, Robert Pieper, Jürgen Zentek

**Affiliations:** Institute of Animal Nutrition, Freie Universität Berlin, Koenigin-Luise-Straße 49, 14195 Berlin, Germany

## Abstract

In this study, the effect of different DNA extraction procedures and primer sets on pyrosequencing results regarding the composition of bacterial communities in the ileum of piglets was investigated. Ileal chyme from piglets fed a diet containing different amounts of zinc oxide was used to evaluate a pyrosequencing study with barcoded 16S rRNA PCR products. Two DNA extraction methods (bead beating versus silica gel columns) and two primer sets targeting variable regions of bacterial 16S rRNA genes (8f-534r versus 968f-1401r) were considered. The SEED viewer software of the MG-RAST server was used for automated sequence analysis. A total of 5.2 × 10^5^ sequences were used for analysis after processing for read length (150 bp), minimum sequence occurrence (5), and exclusion of eukaryotic and unclassified/uncultured sequences. DNA extraction procedures and primer sets differed significantly in total sequence yield. The distribution of bacterial order and main bacterial genera was influenced significantly by both parameters. However, this study has shown that the results of pyrosequencing studies using barcoded PCR amplicons of bacterial 16S rRNA genes depend on DNA extraction and primer choice, as well as on the manner of downstream sequence analysis.

## 1. Introduction

Molecular tools such as the recently introduced method of massively parallel sequencing (deep sequencing) [1, 2] greatly facilitate the study of complex bacterial communities and provide deep insights into their compositions [3–5]. Combined with the technique of barcoded PCR amplicons, deep sequencing methods are able to process many samples at a relatively low cost per sequence [6, 7]. Deep sequencing is, therefore, a promising tool for examining the influence of nutritional and other factors on intestinal microbial communities and functionalities.

However, as with any new technology, pitfalls exist. For barcoded PCR amplicon sequencing studies, nucleic acids must be extracted and the resulting DNA extract should ideally represent the entire bacterial diversity in a given habitat. Furthermore, barcoding requires a PCR step, which depends on primers that should ideally cover the complete bacterial diversity. Finally, the evaluation of sequence reads is based on databases, most of which are not yet suited for massive sequence inputs [8] and sequence quality is often found to be suboptimal [9, 10].

In regard to DNA extraction from complex samples, a multitude of studies have reported that any given nucleic acid extraction method is biased towards certain bacterial groups [11–13]. Complex samples such as environmental samples from soil, waste treatment, or the gastrointestinal tract harbour not only diverse microbial communities, but also other components including mixtures of different carbohydrates, proteins, or minerals. Bacteria can adhere to these compounds and are, thus, more difficult to extract than from culture media. Additionally, substances that are chemically related to nucleic acids such as polyphenolic substances (humic acids and certain components of dietary fibre) can be coextracted and act as powerful PCR inhibitors [14]. Gram-positive cell walls are generally more rigid than gram-negative cell walls, and the extraction of bacterial DNA itself, therefore, becomes a balance between efficient cell lysis and the destruction of DNA from already lysed cells. The most efficient rupturing of bacterial cell walls seems to be achieved by bead beating [13], although commercial kits such as the QIAGEN stool amp kit yield high amounts of stool DNA without bead beating [12].

The correct choice of primer binding site is naturally of primary interest for any PCR based study. For barcoded PCR amplicons, DNA must be amplified in order to sequence multiple samples in a single pyrosequencing run. The hypervariable regions of the bacterial 16S rRNA gene are generally the targets of choice, as the 16S rRNA gene is a valuable phylogenetic marker but also has the advantage of being the most sequenced bacterial gene; that is, sequences from pyrosequencing studies can be assigned against a large collection of reference sequences. However, it has been shown that there is no universal primer set that covers all known bacterial 16S rRNA genes [15–17].

The aim of this study was, therefore, to evaluate the impact of DNA extraction, primer sets, and automated data evaluation on final results.

## 2. Methods

### 2.1. Samples

The study was approved by the local state office of Health and Social Affairs “Landesamt für Gesundheit und Soziales, Berlin” (LaGeSo Reg. No. 0347/09).

A total of 12 ileal samples from 40- to 42-day-old piglets fed a standard starter diet supplemented with 200 or 3000 mg g^−1^ ZnO (*n* = 6 per group), respectively, were used for this study. Subsequent to the euthanasia of the piglets, the gastrointestinal tracts were opened immediately, and the contents of the ileum were removed and stored at −80°C.

### 2.2. DNA Extraction

#### 2.2.1. Procedure I

Total nucleic acids were extracted from 1 g of ileal digesta by using a Guanidinium thiocyanate (4 M) containing lysis buffer at 90°C for 2x 5 minutes, 2x 1 minute bead beating with acid washed glass beads (*∅* 0.3–0.5 mm), subsequent phenol/chloroform (50 : 50, v/v) extraction, and isopropanol (98%) precipitation. Crude extracts were purified to PCR grade DNA with commercial silica gel spin columns (NucleoSpinKit Tissue, Machery-Nagel, Dueren, Germany). The amount of DNA was measured with fluorescence using SYBR green I and calf thymus DNA as reference DNA.

#### 2.2.2. Procedure II

DNA extraction was performed with a commercial kit (Qiagen Stool kit, Qiagen, Hilden, Germany) and 200 mg ileal digesta in triplicate according to the instructions of the manufacturer except for an increase in temperature during the lysis step to 90°C. Purified DNA was then pooled per sample and the DNA was quantified as described above.

### 2.3. Preparation of Sequencing PCR Amplicons

DNA samples were diluted to 100 ng *μ*L^−1^, and 1 *μ*L was used in triplicate for 25 *μ*L PCR reactions. Two primer sets (S-D-Bact-0008-a-S-20/S-D-Bact-0534-a-A-17 and S-D-Bact-0968-a-S-18/S-D-Bact-1401-a-A-17) at a concentration of 0.3 *μ*M were used to amplify two regions of bacterial 16S rRNA genes. Primers were tagged with unique hexamer nucleotides in order to sort PCR products after sequencing (supporting information, Table S1). A commercial master mix kit (HotStarTaq Plus Master Mix; Qiagen, Hilden; with added SYBR green I during cycle number optimization) was used for PCR amplification under the following cycling conditions: 1x 15 min at 95°C, 32x (for the 8f-534r set) or 35x (for the 968f-1401r set) 15 sec at 95°C, 30 sec at 55°C, 30 sec at 72°C, and 1x 1 min 20°C. Optimal amplification conditions were defined for each primer combination by the cycle number before the real time PCR amplification curves entered a plateau with no further increase of total fluorescence. Cycling was performed on a Stratagene MX3000p (Stratagene, Amsterdam, The Netherlands). PCR products were removed immediately after the last cycle and stored at −20°C until further analysis.

The PCR products were purified with a commercial kit (Qiaquick nucleotide removal kit, Qiagen, Hilden, Germany) and the amount of DNA was determined as described above. Equimolar dilutions of all samples were then combined into one master sample per extraction procedure.

### 2.4. Pyrosequencing Procedures

Pyrosequencing was performed by AGOWA (Berlin, Germany) on a Genome Sequencer FLX system using a Titanium series PicoTiterPlate, which was split in half to accommodate the two DNA master samples from different extraction procedures.

### 2.5. Processing and Phylogenetic Assignment of Sequence Reads

Sequence reads were sorted according to barcodes and primer combination, resulting in 48 single data files. After removal of the sample barcodes and primer sequences, data files were uploaded to the MG-RAST server [18, 19] and processed by its SEED software using SILVA SSU [20] as reference databases.

The phylogenetic profile of each sample was computed with the following parameters from the SEED software: maximum *e*-value of 1*e*-5, minimum percent identity of 98%, and minimum alignment length of 150 bases. Sequences that were assigned as unclassified or of eukaryotic origin were not considered in the analysis process.

For statistical interpretation, the next step in the analysis was the deletion of all data with four or less identical sequence reads per sample in order to increase the confidence of sequence reads and to reduce the bias through possible sequencing errors [21, 22]. Also, sequence reads that only occurred in one sample were deleted in order to focus on more common bacterial species. The remaining sequences were used to calculate the relative abundance of specific sequence reads in a sample. These percentages were then used for further statistical analysis.

### 2.6. Statistical Analysis

Arithmetic means and standard errors were calculated for all parameters. ANOVA-procedures were carried out with the software SPSS 15.0 after using the Levene test for homogeneity of variances to determine significant differences at the 0.05 level. Data that failed the homogeneity of variance test was analyzed with the nonparametric Mann-Whitney *U* test to determine asymptotic significant differences. Multiple comparisons of data without homogenous variances were performed using the Tamhane test. Furthermore, data groups with only one data point were omitted to allow multiple comparisons for the remainder of the data groups.

## 3. Results

### 3.1. Yield and Length of Sample Tag PCR Products

DNA extraction, subsequent barcode PCR, and merging of 12 PCR products per extraction procedure yielded two master sample pools of 30 and 50 ng *μ*L^−1^, respectively. The length of PCR products as determined by agarose gel electrophoresis was 438–532 bp for the extraction procedure I and 524–608 bp for the extraction procedure II.

### 3.2. 454-Pyrosequencing Statistics

The 454-sequencing of two master samples yielded a total of 1.11 × 10^6^ sequences with an average read length of 379 bases. After the correction for read length (minimum 150 bases), 6.05 × 10^5^ sequence reads were used for further analysis. There were no significant differences between dietary treatments, but high individual variation was observed. On average, 24763 (±19867) and 26092 (±18054) sequence reads were present in the 200 mg g^−1^ ZnO and 3000 mg g^−1^ ZnO experimental group, respectively.

### 3.3. Distribution of Read Length and GC Content

**Figure 1:**
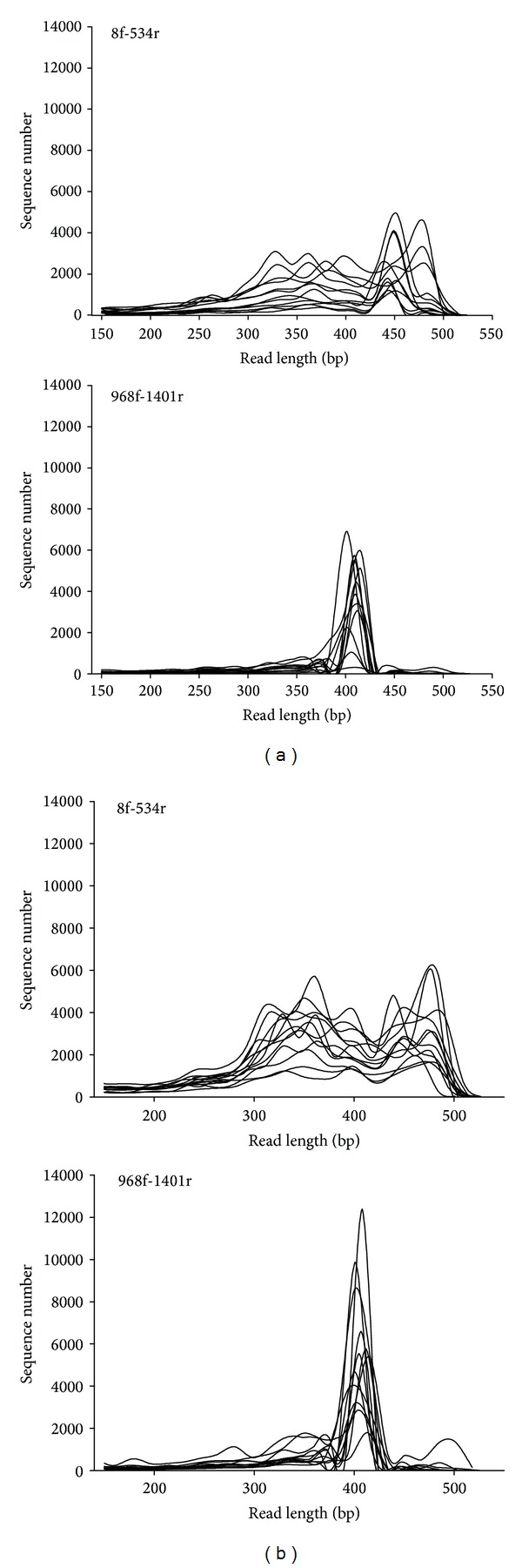
Set of curves for sequence length of single samples (*n* = 12); (a) = extraction I; (b) = extraction II.

Figure 1 shows sets of curves on the distribution of length of sequence reads for single samples. The extraction procedures did not differ in the distribution of sequence length. The primer set 8f-534r led to a more broadly distributed proportion of sequence length with a higher proportion of sequences around 300–400 bases and peaks for some samples at 450 and 480 bases, respectively. In contrast, the primer set 968f-1401r displayed a sharp peak of sequence length at around 400–430 bases for all samples.

**Figure 2:**
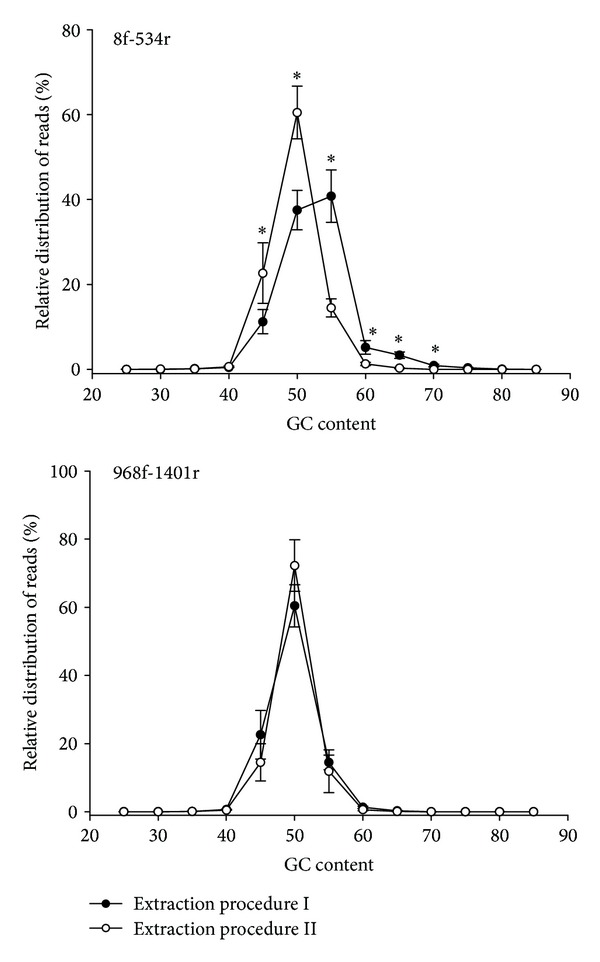
GC content of sequence reads for two extraction procedures and primer sets. ∗ = significantly different at the respective GC content (*P* ≤ 0.05).

The GC content of the sequence reads is shown in Figure 2. There were no significant differences for the primer set 968f-1401r for both extraction procedures. However, the primer set 8f-534r led to a significant shift to more GC-rich sequences, when the extraction procedure I was used.

### 3.4. Exclusion of Sequences Assigned as “Unclassified” and Low Occurrence Reads

The percentage of unclassified/uncultured sequences split by extraction procedure and primer set is shown in Table S2, Supplementary Material available online at http://dx.doi.org/10.1155/2014/548683. Unclassified sequences in the SILVA database ranged from 0.2 to 6.2% of total sequences, which were mostly assigned to unclassified Clostridiales (data not shown).

After exclusion of unclassified/eukaryotic sequences as well as filtering for minimum occurrence (5 sequences), 4.2 × 10^5^ sequences remained for further analysis.

### 3.5. Influence of the Extraction Procedure and Primer Choice on Total Number of Sequences and Assigned Bacterial Genera

Table 1 shows the total number of aligned sequences for both extraction procedures and primer sets, as well as the total number of assigned bacterial genera after filtering for low sequence occurrence. A multifactorial ANOVA analysis of the data is shown in Table 2.

The extraction procedure II proved to be superior in terms of total sequence reads and number. The mean number of the assigned bacterial genera was significantly different. Regarding the primer sets, the 8f-534r primer set generally led to more sequence reads and detected more bacterial genera compared to the 968f-1401r primer set with the extraction procedure II. The removal of sequences with less than five reads per sample reduced the total amount of sequence reads only slightly, whereas a drastic reduction in assigned bacterial genera was observed (see supporting information for data on unprocessed sequences, Table S3).

However, the multivariate ANOVA analysis revealed that there were highly significant interactions for extraction procedure and the choice of the primer set.

### 3.6. Influence of the Extraction Procedure and Primer Sets on Phylogenetic Assignments

Significant differences were observed for most bacterial orders depending on the choice of the extraction procedure or primer set.  Table 3 shows the relative distribution of the 12 most prominent bacterial orders from a total of 25 orders that were detected. The Lactobacillales order showed the highest amount of assigned sequence reads for all tested parameters, followed by Clostridiales and Enterobacteriales/Actinomycetales. The extraction procedure II showed numerically higher Lactobacillales reads than the extraction procedure I. The 8f-534r primer set also had numerically higher amounts of Lactobacillales reads than the 968f-1401r set, but significant differences were only found for the combination of the extraction procedure I and primer set 968f-1401r. On the contrary, the extraction procedure I generally led to a significantly higher relative abundance of Clostridiales reads. Similarly, the primer set 968f-1401r was superior for the detection of Clostridiales. Very high numerical differences regarding the extraction procedure were also found for the order Enterobacteriales, although no significant differences were observed due to very high individual variations.

Other bacterial orders were also influenced by either extraction procedure or primer set. Thus, Actinomycetales, Bacilliales, Fusobacteriales, Erysipelotrichales, and Caulobacteriales seemed to be extracted more effectively by the extraction procedure I, while Pseudomonadales, Campylobacterales, and Neisseriales were detected more effectively by the extraction procedure II. Similarly, Fusobacteriales, Burkholderiales, and Campylobacterales assignments were more pronounced with the primer set 8f-534r, whereas more Actinomycetales and Bacilliales sequence reads were detected with the primer set 968f-1401r.

On the genus level, a total of 154 bacterial genera were detected in processed sequence reads with the two extraction procedures and the two primer sets. Of the total number of genera, 101 bacterial genera were detected by the SILVA database.

Table 4 shows the relative distribution of the major bacterial genera, which exceeded 0.1% of total reads in the database. Regarding the extraction procedure, the combined amount of major genera for both primer sets was 26.8 (±9.4) for the extraction procedure I versus 22.3 (±2.3) genera for the extraction procedure II. The most prominent differences between both extraction procedures were observed for genera of the Clostridiales order, in which the extraction procedure II led to fewer genera above 0.1% of the total sequences. According to the ANOVA analysis, the following genera were significantly influenced by the extraction procedure:* Aerococcus* spp.,* Clostridium* spp.,* Enterococcus* spp.,* Leuconostoc* spp.,* Microbacterium* spp.,* Neisseria* spp.,* Sarcina* spp.,* Staphylococcus* spp.,* Streptococcus* spp.,* Veillonella* spp., and* Weissella* spp. Regarding the primer sets, the combined amount of major genera for both extraction procedures was 22.8 (±4.3) genera for primer set 8f-534r versus 26.7 (±12.0) genera for primer set 968f-1401r. Drastic differences were observed regarding the percentage of assigned bacterial genera. For instance, the 8f-534r primer set led to an average of 65.5%* Lactobacillus* spp. sequences, while the 968f-1401r primer set only displayed an average of 25.4%. On the contrary, the 8f-534r primer set only resulted in an average of 9.6%* Sarcina *spp. sequences, while the 968f-1401r primer set showed 22.1%. In detail, the primer set 8f-534r yielded significantly higher percentages for* Bacillus* spp.,* Fusobacterium* spp.,* Lactobacillus* spp.,* Lactococcus* spp., and* Streptococcus* spp., whereas the primer set 968f-1401r showed higher percentages for* Clostridium* spp.,* Gemella* spp.,* Lachnospira* spp.,* Leuconostoc* spp.,* Microbacterium* spp.,* Sarcina* spp., and* Weissella* spp. Contradicting results were observed for* Macrococcus* spp., which showed higher percentages with the 8-f-534r primers using the extraction method I, but the primer set 968f-1401r showed higher percentages with the extraction method II.

### 3.7. Comparative Data Analysis among Experimental Groups

Table 5 shows richness, Shannon index, and evenness of the sequence data sorted by experimental group of piglets. The combined data evaluation for extraction procedures showed no significant differences in species richness for extraction procedure I, but richness increased in animals fed the high dietary zinc oxide concentration. Vice versa, evenness was not significantly different for extraction procedure II, but it showed increased evenness for the higher dietary zinc oxide concentration. Combined data of the Shannon index led to significant increases for both extraction procedures. The same pattern was seen for combined data of primer sets.

The evaluation of data comprising extraction procedures and primer sets showed a numerical decrease in species richness for data from extraction procedure I and primer set 8f-534r, but the opposite was true for data from extraction procedure II and primer set 968f-1401r. Similarly, the Shannon index failed to reach significant difference among experimental groups for data from extraction procedure I and primer set 8f-534r. Data from extraction procedure II and both primer sets yielded significant increases in species richness for animals fed the high dietary zinc oxide concentration, but no significant differences were observed for Shannon index and evenness.

Comparative results for different extraction procedures and primer sets were also observed for many genera (see supporting information, Table S4). The relative sequence abundance of* Clostridium* spp.,* Dorea* spp.,* Gemella* spp.,* Leuconostoc* spp.,* Microbacterium* spp.,* Peptostreptococcus* spp.,* Rhodococcus* spp.,* Sarcina* spp.,* Streptococcus* spp.,* Veillonella *spp., and* Weissella* spp. was numerically or significantly different for one or more of the studied factors. As examples, for* Clostridium* spp. using extraction procedure II, primer set 968f-1401r led to a numerical decrease. Using extraction procedure I, primer set 968f-1401r for* Microbacterium* spp., the database showed almost identical relative sequence abundance. Although the trend for increasing or decreasing relative sequence abundance was often similar among experimental groups, percentages differed for many combinations of extraction procedures and primer sets. As an example, if one would use extraction procedure I, the primer set 8f-534r would show a drastic and significant increase for* Streptococcus* spp. and* Leuconostoc* spp. in animals fed the high dietary zinc oxide concentration. If one would have employed the primer set 968f-1401r with the same extraction procedure, only a moderate nonsignificant increase would be detected for these genera.

## 4. Discussion

This study was carried out to investigate the effect of different DNA extraction procedures and primer sets on pyrosequencing results regarding the composition of bacterial communities in the ileum of piglets. Barcoded 16S rRNA PCR amplicons have been employed in many different pyrosequencing studies over the last few years. Thus, the analysis of the microbiota in the gut of humans [23], pigs [24], and rodents [25, 26] as well as the analysis of cattle feces [27], plant viruses [28], forest soil fungi [29], soils [30, 31], hot springs [32], the atmosphere [33], sea food [34], or even human lymphocyte clonality [35] relied on the method of using barcoded primer sets for the detection of microbial communities. Although the barcoded amplicon method undoubtedly reduces the yet expensive use of massively parallel sequencing, no methodological study has been published on pre- and postsequencing parameters to the knowledge of the authors.

### 4.1. Processing of Sequence Data

The processing of sequence reads for low occurrence seemed justified, as the number of genera in unprocessed sequence data was more than twice as high as in processed sequence data, but the total number of deleted sequences was low. In addition, sequence reads with less than five sequences only occurred in a few samples. Thus, using unprocessed sequence reads would have introduced a bias towards genera of rare occurrence. This was not justified, because it would have distorted a meaningful statistical analysis of the factors studied.

The total number of unclassified sequences was in the range of 0.6% to 6.2% depending on primer set and extraction procedure which was considered as low and not contributing to the goals of this study.

### 4.2. Extraction Procedures

It is known that the yield of genomic DNA from bacterial species depends on the type of extraction procedure employed [12, 13]. Although the total DNA content of the master samples was very similar, the commercial silica-gel based extraction procedure led to approximately 3- to 5-fold higher numbers of total sequence reads than the bead beating method. Bead beating may have disrupted plant material from feed and, thus, more plant derived PCR inhibitors may have been present in subsequent DNA extracts. In fact, a longer amplification (3 cycles) was observed during PCR optimization to reach a plateau for the 968f-1401r primer set compared to the 8f-534r primer set. However, as diluted PCR amplicons were used to generate the master samples for sequencing, the lower sequencing yield with the bead beating procedure cannot be related to the presence of PCR inhibitors in the original DNA extracts. A reduced sequence yield could also originate from poor quality of the PCR amplicons, which would lead to a reduced sequence yield in the DNA library after processing (blunt end preparation, ligation PCR), but read lengths were very similar for both extraction procedures. Finally, DNA determination of the master samples may have been incorrect. DNA determination was carried out with calf thymus DNA as reference DNA. Calf thymus DNA has a GC content of only 42%, but PCR amplicons from the bead beating procedure and the primer set 8f-534r led to PCR amplicons with a GC content of 50–55%. This combination generally also produced a twofold higher sequence yield than the 968f-1401r primers, which displayed the majority of sequences at 45–50% GC. It is known that minor groove binding dyes such as SYBR green I depend on GC content [36, 37] and, thus, the higher GC content of PCR amplicons produced by the bead beating procedure may have led to an underestimation of the true DNA content.

The extraction procedures differed in extraction efficiency regarding bacterial order and genera. The distribution of sequence reads between different bacterial orders was more uniform for the bead beating procedure than for the commercial extraction kit, because significantly higher proportions of the dominant Lactobacillales were prevalent in DNA extracts from the commercial extraction kit regardless of the chosen primer set.

No clear distinction could be found between the more rigid gram-positive bacteria and the gram-negative bacteria, which have been reported to be easier to extract, as both extraction procedures differed in yields for several gram-positive (Lactobacillales versus Clostridiales) and gram-negative orders (Enterobacteriales versus Pseudomonadales). However, in regard to bacterial genera known to adhere to intestinal epithelial cells or mucus, some differences were observed. Thus, with the exception of Campylobacterales (mainly Arcobacter), the bead beating method was superior for Enterobacteriales (mainly Klebsiella), Actinomycetales (Actinomyces), Fusobacteriales (mainly Fusobacterium), Neisseriales (mainly Neisseria), and Erysipelotrichales (only Erysipelothrix). All the mentioned bacterial genera contain species that are known to adhere strongly to epithelial cells or mucus [38–41]. Although the commercial extraction procedure yielded a higher percentage for the dominating Lactobacillales, among which* Lactobacillus* spp. has a known adherence potential, the most pronounced differences regarding extraction procedures were found for* Weissella* spp., which are not known to adhere to epithelial cells. Epithelial cells and mucus are shed continuously in the proximal parts of the small intestine and bacteria that adhere to epithelial cells are likely to be present in ileum digesta. Thus, the thorough physical disruption of particles by bead beating may have enhanced the extraction of bacterial cells adhering to intestinal epithelial mucus or feed particles. Finally, the enhanced detection of genes for 16S rRNA chloroplasts from plants such as the major diet components soy and wheat indicates that the bead beating procedure successfully disrupted plant cell walls and must, therefore, be considered as the more thorough method regarding disintegration of sample particles.

Both extraction procedures displayed similar total number of genera and diversity indices. However, considering only the dominant bacterial genera above 0.1% of total sequences per sample, especially genera of the Clostridiales order were better represented by the bead beating method and, thus, the richness (amount of genera) of dominant bacteria was higher. This has implications for barcoding pyrosequencing studies which cover high sample numbers, because less barcoded PCR amplicons per sample will be detected and, therefore, dominant bacteria will play a larger role in determining the bacterial composition. In conclusion, DNA extraction procedures with bead beating seem to be superior, but due to the strong disintegration of particles by bead beating, removal of PCR inhibitors must be complete.

### 4.3. Primer

In contrast to sequencing genomic DNA of a few samples without any amplification, barcodes can be used in pyrosequencing studies to drastically increase the amount of samples on a single pyrosequencing plate. The drawback of the ability to sequence multiple samples is that an additional PCR is required for each sample in order to apply the respective tags to each PCR product. This procedure requires primer sets that naturally introduce a bias for the subsequent sequence analysis. This study used four commonly implemented primers that target the hypervariable regions V1-V3 (8f-534r) and V6-V8 (968f-1401r) of bacterial 16S rRNA genes. The results show significant differences of read percentages on the order and genus level. Thus, of the major orders, Lactobacillales, Fusobacteriales, Burkholderiales, and Campylobacterales rRNA genes were better amplified by primers spanning the V1-V3 region, whereas Clostridiales, Actinomycetales, Bacilliales, and Neisseriales were better represented by primers spanning the V6-V8 region. No differences were observed for Enterobacteriales, Pseudomonadales, Erysipelotrichales, or Caulobacteriales.

Even within the dominant Lactobacillales, significant and varying influences of primer sets were observed for four of seven dominant genera (*Lactobacillus *spp.,* Weissella* spp.,* Leuconostoc* spp., and* Enterococcus* spp.). However, amplification of members of the Clostridiales order, which represented the second most abundant order, was more uniform as all genera were best amplified by primers spanning the V6-V8 region. These results confirm data from other studies on the variability of bacterial 16S rRNA gene amplification using “universal” primers for microbial community analysis [17, 42–44].

This primer dilemma may be solved for pyrosequencing studies by using more than one primer pair to cover hypervariable regions of the 16S rRNA gene. The authors have used this approach to study the influence of zinc oxide on porcine ileal bacterial communities [45] by combining sequence reads of the two primer sets used in the present study on the basis of larger sequence number per single samples. It seems to be imperative for the design of barcoded pyrosequencing studies to examine the main bacterial composition in a given habitat in order to choose a primer set that covers most of the bacterial community.

Finally, the primer set targeting the hypervariable regions V1-V3 amplified a considerable proportion of 16S rRNA genes of plant chloroplasts, reducing the amount of sequences of bacterial origin. Although this may not apply to many habitats, all environments that contain significant amounts of plants in form of feed or roots should take notice of the possibility that PCR amplicons resulting from the 8f-534r set could be contaminated with plant chloroplast sequences. Furthermore, deposited sequences attributed to “uncultured Deferribacterales” by databases should be considered with caution, depending on the habitat.

### 4.4. Comparative Data Analysis among Experimental Groups

Many methods that are used for analysis of biological samples from two or more different environments will lead to similar trends although absolute values may differ. According to the results of this study, that statement may not be true for pyrosequencing of barcoded 16S rRNA gene amplicons. Already on the primary methodological level, differences for species richness were observed among the low and high dietary zinc oxide experimental groups depending on the method of DNA extraction. Adding different primer sets to the analysis, one would conclude a nonsignificant decrease for species richness as well as moderate nonsignificant increase for the Shannon index, if bead beating and primer set 8f-534r were used. Using the same DNA extraction method with the primer set 968f-1401r, the observed drastic increase in species richness and Shannon index would lead to the conclusion that dietary zinc oxide has a major impact on bacterial communities in the ileum of piglets.

Even more drastic effects would be generated on the genus level. For* Sarcina* spp., investigators using the commercial spin column method and primer set 8f-534r would not even detect this genus, while the bead beating method would indicate* Sarcina* spp. to be a major component of the bacterial community, which is drastically reduced due to dietary zinc oxide. As this tendency was observed for other genera as well, the biological implications and drawn conclusions may be completely different.

## 5. Conclusions

This empirical study has shown that the choice of extraction procedures and primer can severely influence the outcome of pyrosequencing studies. DNA extraction seemed more complete using bead beating. A viable solution for PCR amplification could be the use of two or more primer sets to completely cover the bacterial diversity in complex samples. With respect to published studies on barcoded pyrosequencing of bacterial 16S rRNA genes, the method and derived results should be regarded with care.

## Supplementary Material

Additional supporting information may be found in the online version of this article:
Table S1: Bar code PCR Primer sequences
Table S2: Percentage of unclassified sequences (n = 12)*[*%*]*
Table S3: Mean number of sequences and bacterial genera of unprocessed sequence data detected by three different data bank alignments (n = 12)

## Figures and Tables

**Table 1:** Mean number of assigned sequences and number bacterial genera detected by three different data bank alignments after filtering for low sequence occurrence^1^ (*n* = 12).

DNA Extraction	Primer set	Sequences	Genera
Procedure I	8f-534r	5004 (±1409)^a^	54 (13.1 ± 2.1)^A^
	968f-1401r	2748 (±493)^a^	54 (15.5 ± 2.6)^A^
Procedure II	8f-534r	16596 (±1790)^b^	55 (23.9 ± 2.7)^B^
	968f-1401r	10257 (±1423)^b^	43 (18.0 ± 1.8)^AB^

*Total and mean genera per sample.

^1^Different superscripts within columns are significantly different (*P* ≤ 0.05; ANOVA, capital letters = Tamhane test).

**Table 2:** *P*-values of a multifactorial ANOVA analysis of total sequence reads and assigned bacterial genera before and after removal of low number sequences (<5).

Factor	Total sequence reads		Removal of low number sequences	
	Sequence number	Genera	Sequence number	Genera
Extraction procedure	0.000	0.003	0.000	0.008
Primer	0.000	0.014	0.000	0.171
Extraction × Primer	0.000	0.001	0.000	0.003

**Table 3:** Influence of extraction procedure and primer sets on the relative distribution of sequence reads for main bacterial orders^1^ [%] (*n* = 12).

Extraction	Primer set	Lactobacilliales	Clostridiales	Enterobacteriales	Actinomycetales	Bacillales	Fusobacteriales
I	8f-534r	**75.9** ** (±8.1)** ^**b**^	**15.2 (±7.4)** ^**ab**^	5.7 (±5.3)	**1.5 (±0.4)** ^**b**^	**0.39 (±0.20)** ^**a**^	**0.19 (±0.12)** ^**b**^
	968f-1401r	**41.9 (±7.8)** ^**a**^	**37.2 (±9.2)** ^**c**^	6.8 (±6.1)	**10.4 (±3.2)** ^**d**^	**1.7 (±0.8)** ^**b**^	**0.10 (±0.05)** ^**ab**^
II	8f-534r	**90.8 (±5.4)** ^**b**^	**7.4 (±5.5)** ^**a**^	0.19 (±0.08)	**0.36 (±0.07)** ^**a**^	**0.15 (±0.06)** ^**a**^	**0.21 (±0.06)** ^**b**^
	968f-1401r	**82.1 (±7.8)** ^**b**^	**16.1 (±7.8)** ^**ab**^	0.21 (±0.06)	**1.1 (±0.18)** ^**b**^	**0.11 (±0.03)** ^**a**^	**0.01 (±0.01)** ^**a**^

Extraction	Primer set	Burkholderiales	Pseudomonadales	Campylobacterales	Neisseriales	Erysipelotrichales	Caulobacterales

I	8f-534r	**0.02 (±0.02)** ^**a**^	n.d.	**0.18 (±0.15)** ^**ab**^	n.d.	n.d.	0.01 (±0.01)
	968f-1401r	**0.06 (±0.05)** ^**a**^	**0.01 (±0.01)** ^**a**^	**0.05 (±0.05)** ^**a**^	**0.88 (±0.44)** ^**b**^	n.d.	n.d.
II	8f-534r	**0.23 (±0.06)** ^**b**^	**0.15 (±0.05)** ^**b**^	**0.19 (±0.06)** ^**b**^	**0.21 (±0.06)** ^**b**^	n.d.	0.002 (±0.001)
	968f-1401r	**0.05 (±0.02)** ^**a**^	**0.20 (±0.06)** ^**b**^	n.d.	**0.15 (±0.05)** ^**ab**^	n.d.	n.d.

n.d.: not detected.

^1^Different superscripts within a column are significantly different (*P* ≤ 0.05; ANOVA).

**Table 4:** Effect of extraction procedure and primer set on the relative distribution of main bacterial genera^1^ (>0.1% of total sequence reads) [% sequence reads].

Extraction	Primer set	Lactobacillus	Weissella	Leuconostoc	Streptococcus	Lactococcus	Aerococcus
I	8f-534r	**70.4 (±7.8)** ^**c**^	**0.36 (±0.18)** ^**a**^	n.d.	**5.1 (±1.2)** ^**ab**^	**0.31 (±0.14)** ^**a**^	0.93 (±0.63)
	968f-1401r	**33.3 (±6.4)** ^**b**^	**3.0 (±1.1)** ^**b**^	**0.89 (±0.31)** ^**a**^	**5.3 (±1.7)** ^**ab**^	0.43	1.0 (±0.5)
II	8f-534r	**59.1 (±8.7)** ^**b**^	**19.3 (±5.0)** ^**c**^	**4.6 (±1.1)** ^**ab**^	**5.8 (±2.8)** ^**b**^	**1.9 (±0.5)** ^**b**^	0.17
	968f-1401r	**14.2 (±5.5)** ^**a**^	**47.6 (±6.3)** ^**d**^	**18.8 (±2.8)** ^**b**^	**1.0 (±0.3)** ^**a**^	**0.34 (±0.06)** ^**a**^	0.21 (±0.03)

Extraction	Primer set	Enterococcus	Sarcina	Clostridium	Lachnospira	Faecalibacterium	Veillonella

I	8f-534r	**1.2 (±0.8)** ^**ab**^	**13.9 (±7.4)** ^**a**^	**0.59 (±0.15)** ^**a**^	0.72 (±0.16)	0.93	1.1 (±0.6)
	968f-1401r	0.54	**33.6 (±9.8)** ^**b**^	**1.7 (±0.5)** ^**ab**^	n.d.	n.d.	0.51 (±0.13)
II	8f-534r	**0.28 (±0.05)** ^**a**^	**6.9 (±5.5)** ^**a**^	**0.25 (±0.07)** ^**a**^	n.d.	n.d.	0.44 (±0.22)
	968f-1401r	0.28	**15.3 (±7.8)** ^**ab**^	**0.51 (±0.14)** ^**a**^	n.d.	n.d.	0.41 (±0.27)

Extraction	Primer set	Eubacterium	Peptostreptococcu	Ruminococcus	Dorea	Megasphaera	Klebsiella

I	8f-534r	0.20	0.31	**0.43 (±0.24)** ^**a**^	n.d.	1.1	11.0 (±10.3)
	968f-1401r	1.8 (±1.2)	**1.1 (±0.8)** ^**b**^	n.d.	**2.0 (±0.9)** ^**ab**^	n.d.	10.9 (±9.9)
II	8f-534r	n.d.	**0.36 (±0.005)** ^**a**^	n.d.	n.d.	n.d.	0.15 (±0.02)
	968f-1401r	0.13 (±0.01)	**0.72 (±0.21)** ^**ab**^	n.d.	n.d.	n.d.	0.14

Extraction	Primer set	Salmonella	Escherichia	Citrobacter	Enterobacter	Pantoea	Microbacterium

I	8f-534r	n.d.	0.47 (±0.22)	n.d.	n.d.	n.d.	**1.2 (±0.3)** ^**a**^
	968f-1401r	0.19	0.93 (±0.80)	n.d.	0.51 (±0.28)	0.39 (±0.04)	**10.3 (±3.2)** ^**b**^
II	8f-534r	7.0 (±5.5)	0.15	0.26 (±0.14)	0.22	n.d.	**0.17 (±0.03)** ^**a**^
	968f-1401r	n.d.	n.d.	0.31 (±0.05)	0.17	n.d.	**1.0 (±0.2)** ^**a**^

Extraction	Primer set	Actinomyces	Rhodococcus	Bacillus	Staphylococcus	Macrococcus	Kurthia

I	8f-534r	0.80 (±0.23)	0.63 (±0.18)	0.50 (±0.14)	**0.25 (±0.06)** ^**a**^	**0.23 (±0.07)** ^**a**^	0.31 (±0.06)
	968f-1401r	0.31 (±0.09)	0.20	0.30 (±0.04)	**2.7 (±1.1)** ^**b**^	**0.2 (±0.003)** ^**a**^	n.d.
II	8f-534r	0.26 (±0.07)	0.14 (±0.03)	0.42 (±0.13)	n.d.	n.d.	n.d.
	968f-1401r	0.33 (±0.18)	n.d.	0.13	**0.17 (±0.02)** ^**a**^	0.10	n.d.

n.d.: not detected.

^1^Different superscripts within a row are significantly different (*P* ≤ 0.05; Tamhane Test).

**Table 5:** Effect of extraction procedure and primer set on the comparative diversity indices of main bacterial genera in the ileum of pigs fed 200 mg g^−1^ or 3000 mg g^−1^ dietary ZnO^1^ (>0.1% of total sequence reads) (*n* = 6 per experimental group).

Extraction	Primer set	Database	Richness		Shannon		Evenness	
			200 mg g^−1^	3000 mg g^−1^	200 mg g^−1^	3000 mg g^−1^	200 mg g^−1^	3000 mg g^−1^
Procedure I			16.3 (±10.6)	19.9 (±9.9)	**0.886 (±0.546)** ^**A**^	**1.241 (±0.602)** ^**B**^	**0.329 (±0.164)** ^**A**^	**0.424 (±0.148)** ^**B**^
Procedure II			**18.5 (±7.6)** ^**A**^	**27.1 (±11.0)** ^**B**^	**0.965 (±0.379)** ^**A**^	**1.144 (±0.351)** ^**B**^	0.340 (±0.138)	0.353 (±0.095)

	8f-534r		20.4 (±10.2)	23.0 (±13.5)	**0.782 (±0.504)** ^**A**^	**1.019 (±0.443)** ^**B**^	**0.255 (±0.137)** ^**A**^	**0.343 (±0.106)** ^**B**^
	968f-1401r		**14.5 (±7.1)** ^**A**^	**24.0 (±7.9)** ^**B**^	**1.068 (±0.386)** ^**A**^	**1.366 (±0.481)** ^**B**^	0.414 (±0.120)	0.434 (±0.134)

Procedure I	8f-534r		18.6 (±11.4)	15.3 (±9.1)	0.750 (±0.578)	0.973 (±0.441)	**0.250 (±0.150)** ^**A**^	**0.376 (±0.094)** ^**B**^
	968f-1401r		**14.1 (±9.5)** ^**A**^	**24.6 (±8.7)** ^**B**^	**1.021 (±0.491)** ^**A**^	**1.508 (±0.632)** ^**B**^	0.408 (±0.140)	0.472 (±0.177)

Procedure II	8f-534r		**22.2 (±8.9)** ^**A**^	**30.8 (±12.9)** ^**B**^	0.814 (±0.433)	1.064 (±0.452)	0.260 (±0.127)	0.309 (±0.109)
	968f-1401r		**14.8 (±3.5)** ^**A**^	**23.4 (±7.3)** ^**B**^	1.115 (±0.247)	1.224 (±0.188)	0.420 (±0.098)	0.396 (±0.053)

^1^Different superscripts within a row (highlighted in bold) are significantly different for the respective diversity index (*P* ≤ 0.05; pairwise Mann-Whitney-*U* Test).
