# Supplementary material for: The Influence of DNA Extraction Procedure and Primer Set on the Bacterial Community Analysis by Pyrosequencing of Barcoded 16S rRNA Gene Amplicons
Source: Mol Biol Int. 2014 Jul 10;2014:548683. doi: 10.1155/2014/548683 (PMC4120916; doi:10.1155/2014/548683)
Supplement: Supplementary file 1 — Additional supporting information may be found in the online version of this article: Table S1: Bar code PCR Primer sequences Table S2: Percentage of unclassified sequences (n = 12)[%] Table S3: Mean number of sequences and bacterial genera of unprocessed sequence data detected by three different data bank alignments (n = 12) [file 548683.f1.doc]

**Table S1.** Bar code PCR Primer sequences

| **Name** | **Sequence (5`-3`)** |
| --- | --- |
| F-968-1 | ACACACGAACGCGAAGAACCTTAC |
| F-968-2 | ACATGCGAACGCGAAGAACCTTAC |
| F-968-3 | ACGTACGAACGCGAAGAACCTTAC |
| F-968-4 | AGCAGCGAACGCGAAGAACCTTAC |
| F-968-5 | TGCGCGGAACGCGAAGAACCTTAC |
| F-968-6 | TGTGTGGAACGCGAAGAACCTTAC |
| F-968-7 | GCGTCTGAACGCGAAGAACCTTAC |
| F-968-8 | GTCTCTGAACGCGAAGAACCTTAC |
| F-968-9 | GCTGCTGAACGCGAAGAACCTTAC |
| F-968-10 | GACGCTGAACGCGAAGAACCTTAC |
| F-968-11 | GTGACTGAACGCGAAGAACCTTAC |
| F-968-12 | GATGATGAACGCGAAGAACCTTAC |
| R-1401-1 | ACACACGCGTGTGTACAAGACCC |
| R-1401-2 | ACATGCGCGTGTGTACAAGACCC |
| R-1401-3 | ACGTACGCGTGTGTACAAGACCC |
| R-1401-4 | AGCAGCGCGTGTGTACAAGACCC |
| R-1401-5 | TGCGCGGCGTGTGTACAAGACCC |
| R-1401-6 | TGTGTGGCGTGTGTACAAGACCC |
| R-1401-7 | GCGTCTGCGTGTGTACAAGACCC |
| R-1401-8 | GTCTCTGCGTGTGTACAAGACCC |
| R-1401-9 | GCTGCTGCGTGTGTACAAGACCC |
| R-1401-10 | GACGCTGCGTGTGTACAAGACCC |
| R-1401-11 | GTGACTGCGTGTGTACAAGACCC |
| R-1401-12 | GATGATGCGTGTGTACAAGACCC |
| 8f-1 | TGACACAGAGTTTGATCCTGGCTCAG |
| 8f-2 | TGATGCAGAGTTTGATCCTGGCTCAG |
| 8f-3 | TGCATCAGAGTTTGATCCTGGCTCAG |
| 8f-4 | CTCTGTAGAGTTTGATCCTGGCTCAG |
| 8f-5 | CGATGTAGAGTTTGATCCTGGCTCAG |
| 8f-6 | GTAGCTAGAGTTTGATCCTGGCTCAG |
| 8f-7 | GAGTATAGAGTTTGATCCTGGCTCAG |
| 8f-8 | GTATATAGAGTTTGATCCTGGCTCAG |
| 8f-9 | GTCGATAGAGTTTGATCCTGGCTCAG |
| 8f-10 | GCAGATAGAGTTTGATCCTGGCTCAG |
| 8f-11 | GTGCATAGAGTTTGATCCTGGCTCAG |
| 8f-12 | CGTCGTAGAGTTTGATCCTGGCTCAG |
| 534r-1 | TGACACATTACCGCGGCTGCTGG |
| 534r-2 | TGATGCATTACCGCGGCTGCTGG |
| 534r-3 | TGCATCATTACCGCGGCTGCTGG |
| 534r-4 | CTCTGTATTACCGCGGCTGCTGG |
| 534r-5 | CGATGTATTACCGCGGCTGCTGG |
| 534r-6 | GTAGCTATTACCGCGGCTGCTGG |
| 534r-7 | GAGTATATTACCGCGGCTGCTGG |
| 534r-8 | GTATATATTACCGCGGCTGCTGG |
| 534r-9 | GTCGATATTACCGCGGCTGCTGG |
| 534r-10 | GCAGATATTACCGCGGCTGCTGG |
| 534r-11 | GTGCATATTACCGCGGCTGCTGG |
| 534r-12 | CGTCGTATTACCGCGGCTGCTGG |

**Table S2**. Percentage of unclassified sequences^1^ (n = 12) [%]

| **Database** | **DNA Extraction** | **Primer set** | **Unclassified sequences [%]** |
| --- | --- | --- | --- |
| RDP | Procedure I | 8f-534r | 48.3 (±7.8)^e^ |
|  |  | 968f-1401r | 23.6 (±4.9)^d^ |
|  | Procedure II | 8f-534r | 10.1 (±1.8)^c^ |
|  |  | 968f-1401r | 2.9 (±0.4)^b^ |
| Green Gene | Procedure I | 8f-534r | 2.4 (±1.1)^b^ |
|  |  | 968f-1401r | 6.2 (±1.9)^bc^ |
|  | Procedure II | 8f-534r | 0.2 (±0.1)^a^ |
|  |  | 968f-1401r | 0.6 (±0.1)^a^ |
| SILVA | Procedure I | 8f-534r | 2.2 (±0.4)^b^ |
|  |  | 968f-1401r | 3.5 (±0.6)^b^ |
|  | Procedure II | 8f-534r | 2.6 (±0.4)^b^ |
|  |  | 968f-1401r | 1.2 (±0.3)^ab^ |

^1^ = Different superscripts are significantly different (p≤ 0.05; ANOVA)

**Table S3.** Mean number of sequences and bacterial genera of unprocessed sequence data detected by three different data bank alignments^1^ (n = 12)

| DNA Extraction | Primer set | **RDP** | | **Greengene** | | **SILVA** | |
| --- | --- | --- | --- | --- | --- | --- | --- |
|  |  | Sequences | Genera ^*^ | Sequences | Genera | Sequences | Genera |
| Procedure I | 8f-534r | 8310 (± 2498)^a^ | 131 (46.0 ±5.0)^A^ | 8526 (± 2515)^AB^ | 148 (53.5 ±5.9)^A^ | 5045 (±1418) | 128 (36.6 ±4.3)^A^ |
|  | 968f-1401r | 4286 (± 625)^a^ | 131 (47.8 ±4.5)^A^ | 4851 (± 656)^A^ | 152 (55.6 ±5.2)^A^ | 2788 (±494) | 140 (40.0 ±4.8)^A^ |
| Procedure II | 8f-534r | 25089 (± 3000)^b^ | 171 (62.8 ±6.1)^B^ | 25049 (± 3018)^C^ | 184 (71.2 ±6.8)^B^ | 16653 (±1792) | 163 (57.5 ±5.5)^B^ |
|  | 968f-1401r | 11194 (± 1472)^ab^ | 122 (44.9 ±3.2)^A^ | 9128 (± 1211)^B^ | 152 (52.9 ±4.3)^A^ | 10252 (±1417) | 121 (43.1 ±2.8)^A^ |

^*^ Total and mean genera per sample

^1^ = Different superscripts within columns are significantly different (p≤ 0.05; ANOVA, capital letters = Tamhane test)

**Table S4.** Effect of extraction procedure, primer set and reference data base on comparative results for some main bacterial genera in the ileum of pigs fed 200 mg g-1 or 3000 mg g-1 dietary ZnO1 (> 0.5% of total sequence reads) (n = 6 per experimental group)

| Extraction | Primer | Database | Trial group [mg g^-1^ ZnO] | Clostridium | Dorea | Gemella | Leuconostoc | Microbacterium | Peptostreptococcus | Rhodococcus | Sarcina | Streptococcus | Veillonella | Weissella |
| --- | --- | --- | --- | --- | --- | --- | --- | --- | --- | --- | --- | --- | --- | --- |
| I |  |  | 200 | **0.58 (±0.81)^A^** | 0.63 (±2.03) | n.d. | **0.04 (±0.08)^A^** | 2.7 (± 6.9) | **0.17 (±0.58)^A^** | 0.07 (±0.12) | **28.6 (±34.6)^B^** | 4.65 (±7.61) | **0.32 (±0.64)^B^** | **0.37 (± 0.94)^A^** |
|  |  |  | 3000 | **2.4 (±4.7)^B^** | 0.79 (±2.06) | 0.21 (±0.47) | **0.27 (±0.54)^B^** | 2.8 (±5.6) | **0.60 (±1.33)^B^** | 0.22 (±0.40) | **12.7 (±23.3)^A^** | 4.94 (±4.38) | **0.05 (±0.10)^A^** | **2.26 (±4.58)^B^** |
| II |  |  | 200 | **0.16 (± 0.31)^A^** |  | **0.28 (± 0.89)^A^** | **9.6 (±10.3)^A^** | 0.47 (±0.71) | 0.07 (±0.28) | 0.003 (±0.02) | 13.1 (±25.4) | **1.2 (±1.2)^A^** | 0.28 (±0.50) | **21.8 (±20.6)^A^** |
|  |  |  | 3000 | **0.25 (±0.39)^B^** | 0.04 (±0.12) | **0.38 (±0.54)^B^** | **15.8 (±11.5)^B^** | 0.32 (±0.56) | 0.04 (±0.11) | 0.01 (±0.04) | 1.5 (±3.8) | **4.9 (±7.9)^B^** | 0.08 (±0.08) | **35.8 (±22.4)^B^** |
|  | 8f-534r |  | 200 | 0.19 (±0.31) | 0.06 (±0.26) | 0.03 (±0.10) | 2.0 (±2.8) | **0.11 (±0.20)^A^** | 0.02 (±0.07) | 0.05 (±0.10)^A^ | 9.49 (±25.2) | **3.3 (±5.7)^A^** | 0.42 (±0.73) | 5.7 (±8.3) |
|  |  |  | 3000 | 0.37 (±0.60) | 0.02 (±0.10) | 0.09 (±0.20) | 3.2 (±5.4) | **0.47 (±0.78)^B^** | 0.01 (±0.06) | 0.24 (±0.39)^B^ | 2.5 (±4.4) | **7.2 (±7.4)^B^** | 0.06 (±0.07) | 10.2 (±16.9) |
|  | 968f-1401r |  | 200 | **0.56 (±0.82)^A^** | 0.56 (±2.0)^A^ | **0.25 (±0.89)^A^** | **7.7 (±11.4)^A^** | 3.1 (±6.8) | **0.22 (±0.63)^A^** | 0.02 (±0.08) | 32.2 (±32.6)^B^ | 2.6 (±5.7) | 0.17 (±0.31) | **16.5 (±23.1)^A^** |
|  |  |  | 3000 | **2.3 (±4.7)^B^** | 0.81 (±2.1)^B^ | **0.51(± 0.64)^B^** | **12.8 (±13.4)^B^** | 2.7 (±5.7) | **0.63 (±1.3)^B^** | n.d. | 11.7 (±23.6)^A^ | 2.7 (±4.2) | 0.07 (±0.11) | **28.0 (±25.5)^B^** |
| I | 8f-534r |  | 200 | 0.28 (±0.36) | 0.12 (±0.36) | n.d. | n.d. | **0.17 (±0.26)^A^** | 0.02 (±0.07) | **0.09 (±0.13)^A^** | 18.8 (±33.4) | **4.8 (±7.7)^A^** | **0.41 (±0.82)^B^** | 0.07 (±0.20) |
|  |  |  | 3000 | 0.61 (±0.76) | 0.05 (±0.14) | 0.04 (±0.10) | n.d. | **0.90 (±0.93)^B^** | n.d. | **0.45 (±0.47)^B^** | 5.0 (±5.2) | **5.6 (±3.3)^B^** | **0.02 (±0.05)^A^** | 0.05 (±0.08) |
|  | 968f-1401r |  | 200 | **0.88 (±1.0)^A^** | **1.1 (±2.8)^A^** | n.d. | **0.07 (±0.11)^A^** | 5.3 (±9.2) | **0.31 (±0.81)^A^** | 0.05 (±0.11) | **38.4 (±33.8)^B^** | 4.5 (±7.8) | 0.22 (±0.40) | **0.66 (±1.3)^A^** |
|  |  |  | 3000 | **4.1 (±6.2)^B^** | **1.5 (±2.7)^B^** | 0.39 (±0.62) | **0.54 (±0.67)^B^** | 4.8 (±7.5) | **1.2 (±1.7)^B^** | n.d. | **20.4 (±31.0)^A^** | 4.27 (±5.3) | 0.08 (±0.13) | **4.5 (±5.7)^B^** |
| II | 8f-534r |  | 200 | 0.09 (±0.21) | n.d. | 0.06 (±0.13) | 4.0 (±2.8) | 0.05 (±0.10) | 0.02 (±0.07) | 0.01 (±0.02) | n.d. | **1.7 (±1.5)^A^** | 0.44 (±0.65) | 11.4(±8.6) |
|  |  |  | 3000 | 0.13 (±0.25) | n.d. | 0.15 (±0.25) | 6.4 (±6.2) | 0.04 (±0.07) | 0.02 (±0.09) | 0.03 (±0.06) | n.d. | **8.7 (±9.8)^B^** | 0.11 (±0.06) | 20.3 (±19.4) |
|  | 968f-1401r |  | 200 | **0.23 (±0.38)^A^** | n.d. | **0.50 (±1.2)^A^** | **15.3 (±11.9)^A^** | 0.89 (±0.81) | 0.13 (±0.39) | n.d. | **26.1 (±31.2)^B^** | 0.73 (±0.30) | **0.13 (±0.17)^B^** | **32.4 (±23.8)^A^** |
|  |  |  | 3000 | **0.37 (±0.47)^B^** | 0.07 (±0.17) | **0.62 (±0.65)^B^** | **25.1 (±7.1)^B^** | 0.60 (±0.68) | 0.06 (±0.13) | n.d. | **3.0 (±5.0)^A^** | 1.2(±1.6) | **0.05 (±0.08)^A^** | **51.4 (±11.9)^B^** |
| I | 8f-534r | RDP | 200 | 0.20 (±0.28) | 0.17 (±0.42) | n.d. | n.d. | 0.02 (±0.05) | n.d. | 0.09 (±0.10) | 18.8 (±35.3) | 6.7 (±9.6) | 0.59 (±1.10) | 0.10 (±0.19) |
|  |  |  | 3000 | 0.57 (±0.65) | 0.07 (±0.18) | 0.06 (±0.14) | n.d. | 0.42 (±0.67) | n.d. | 0.39 (±0.43) | 4.4 (±4.7) | 5.4 (±3.3) | 0.03 (±0.07) | 0.05 (±0.08) |
|  |  | Green Gene | 200 | 0.29 (±0.47) | 0.20 (±0.50) | n.d. | n.d. | **0.15 (±0.23)^A^** | n.d. | **0.05 (±0.08)^A^** | 16.1 (±35.5) | **4.3 (±8.8)^A^** | 0.12 (±0.22) |  |
|  |  |  | 3000 | 0.93 (±1.01) | 0.07 (±0.16) | 0.05 (±0.12) | n.d. | **0.86 (±0.80)^B^** | n.d. | **0.36 (±0.41)^B^** | 4.2 (±4.6) | **5.6 (±3.6)^B^** | 0.03 (±0.07) | 0.05 (±0.08) |
|  |  | Silva | 200 | 0.36 (±0.36) | n.d. | n.d. | n.d. | 0.34 (±0.34) | 0.05 (±0.13) | 0.14 (±0.18) | 21.4 (±35.7) | 3.5 (±4.7) | 0.53 (±0.91) | 0.12 (±0.30) |
|  |  |  | 3000 | 0.33 (±0.52) | n.d. | n.d. | n.d. | 1.4 (±1.1) | n.d. | 0.59 (±0.60) | 6.5 (±6.5) | 5.8 (±3.5) | n.d. | 0.06 (±0.09) |
|  | 968f-1468r | RDP | 200 | 0.44 (±0.53) | 1.6 (±3.7) | n.d. | 0.07 (±0.11) | **0.11 (±0.27)^A^** | 0.48 (±1.0) | 0.06 (±0.15) | 36.5 (±36.1) | 5.7 (±10.7) | 0.25 (±0.51) | **0.77 (±1.46)^A^** |
|  |  |  | 3000 | 7.3 (±9.3) | 2.5 (±3.7) | 0.76 (±0.77) | 0.55 (±0.65) | **0.45 (±0.39)^B^** | 1.8 (±2.2) | n.d. | 20.6 (±33.0) | 2.1 (±2.8) | 0.07 (±0.12) | **5.6 (±7.6)^B^** |
|  |  | Green Gene | 200 | 0.57 (±0.64) | 1.4 (±3.3) | n.d. | 0.06 (±0.09) | 6.0 (±8.9) | 0.46 (±1.0) | 0.05 (±0.12) | 35.1 (±36.0) | 4.7 (±8.5) | 0.20 (±0.39) | 0.6 (±1.1) |
|  |  |  | 3000 | 3.3 (±4.6) | 1.5 (±2.7) | 0.42 (±0.59) | 0.27 (±0.42) | 3.0 (±2.9) | 1.0 (±1.5) | n.d. | 16.9 (±33.2) | 3.9 (±4.9) | 0.06 (±0.11) | 4.3 (±6.3) |
|  |  | Silva | 200 | 1.6 (±1.3) | 0.38 (±0.92) | n.d. | **0.08 (±0.13)^A^** | 9.6 (±12.4) | n.d. | 0.03 (±0.08) | 43.5 (±35.0) | 2.9 (±3.7) | 0.21 (±0.37) | 0.63 (±1.4)^A^ |
|  |  |  | 3000 | 1.8 (±1.9) | 0.60 (±1.4) | n.d. | **0.81 (±0.87)^B^** | 10.8 (±10.5) | 0.76 (±1.3) | n.d. | 23.7 (±32.4) | 6.7 (±7.1) | 0.11 (±0.17) | 3.4 (±3.3)^B^ |
| II | 8f-534r | RDP | 200 | 0.10 (±0.24) | n.d. | 0.09 (±0.16) | 3.9 (±2.8) | n.d. | n.d. | n.d. | n.d. | **1.7 (±1.5)^A^** | 0.47 (±0.70) | 10.3 (±7.3) |
|  |  |  | 3000 | 0.11 (±0.22) | n.d. | 0.22 (±0.29) | 6.9 (±7.1) | 0.02 (±0.05) | n.d. | 0.02 (±0.06) | n.d. | **7.9 (±9.1)^B^** | 0.12 (±0.07) | 19.4 (±20.4) |
|  |  | Green Gene | 200 | 0.12 (±0.29) | n.d. | 0.09 (±0.16) | 4.1 (±3.3) | 0.06 (±0.11) | n.d. | n.d. | n.d. | **1.8 (±1.8)^A^** | 0.43 (±0.71) | 9.2 (±7.6) |
|  |  |  | 3000 | 0.23 (±0.36) | n.d. | 0.22 (±0.30) | 7.0 (±7.4) | 0.04 (±0.07) | n.d. | 0.02 (±0.06) | n.d. | **8.1 (±9.1)^B^** | 0.12 (±0.07) | 17.5 (±18.9) |
|  |  | Silva | 200 | 0.07 (±0.11) | n.d. | n.d. | 3.8 (±2.8) | 0.10 (±0.12) | 0.05 (±0.13) | 0.02 (±0.04) | n.d. | **1.5 (±1.3)^A^** | 0.42 (±0.67) | 14.6 (±11.1) |
|  |  |  | 3000 | 0.06 (±0.14) | n.d. | n.d. | 5.4 (±4.8) | 0.07 (±0.08) | 0.06 (±0.15) | 0.03 (±0.07) | n.d. | **10.2 (±12.7)^B^** | 0.08 (±0.06) | 23.9 (±21.9) |
|  | 968f-1468r | RDP | 200 | **0.01 (±0.03)^A^** | n.d. | **0.67 (±1.34)^A^** | 13.8 (±10.9) | 0.09 (±0.07) | 0.04 (±0.09) | n.d. | 24.5 (±31.7) | 0.64 (±0.27) | 0.12 (±0.12) | 35.2 (±26.5) |
|  |  |  | 3000 | **0.33 (±0.40)^B^** | 0.09 (±0.17) | **0.79 (±0.48)^B^** | 22.4 (±5.5) | 0.04 (±0.04) | 0.04 (±0.06) | n.d. | 2.8 (±4.8) | 0.70 (±0.92) | 0.05 (±0.09) | 55.7 (±9.8) |
|  |  | Green Gene | 200 | **0.12 (±0.14)^A^** | n.d. | **0.83 (±1.68)^A^** | 17.7 (±15.2) | 1.4 (±0.84) | 0.04 (±0.11) | n.d. | 26.0 (±33.2) | 0.89 (±0.35) | 0.12 (±0.12) | 24.8 (±20.2) |
|  |  |  | 3000 | **0.58 (±0.69)^B^** | 0.12 (±0.23) | **1.1 (±0.70)^B^** | 29.6 (±8.8) | 0.95 (±0.78) | 0.03 (±0.05) | n.d. | 3.7 (±6.5) | 1.6 (±2.2) | 0.06 (±0.10) | 40.4 (±9.0) |
|  |  | Silva | 200 | 0.58 (±0.50) | n.d. | n.d. | 14.3 (±11.1) | 1.2 (±0.60) | 0.30 (±0.67) | n.d. | **27.7 (±34.5)^B^** | 0.66 (±0.27) | 0.15 (±0.26) | 36.9 (±26.7) |
|  |  |  | 3000 | 0.20 (±0.18) | n.d. | 0.01 (±0.02) | 23.3 (±5.3) | 0.80 (±0.63) | 0.12 (±0.21) | n.d. | **2.6 (±4.4)^A^** | 1.2 (±1.6) | 0.04 (±0.06) | 58.2 (±8.9) |

^1^ = Different capital letters indicate significant differences among experimental groups (highlighted in bold) regarding the respective extraction procedure, primer set and database (Mann-Whitney-U Test)
